# Open University Learning Analytics dataset

**DOI:** 10.1038/sdata.2017.171

**Published:** 2017-11-28

**Authors:** Jakub Kuzilek, Martin Hlosta, Zdenek Zdrahal

**Affiliations:** 1 Knowledge Media Institute, The Open University, Walton Hall, Milton Keynes MK7 6AA, UK; 2 CIIRC, Czech Technical University, Zikova street 1903/4, Prague 166 36, Czech Republic

**Keywords:** Statistics, Education, Scientific data, Computer science

## Abstract

Learning Analytics focuses on the collection and analysis of learners’ data to improve their learning experience by providing informed guidance and to optimise learning materials. To support the research in this area we have developed a dataset, containing data from courses presented at the Open University (OU). What makes the dataset unique is the fact that it contains demographic data together with aggregated clickstream data of students’ interactions in the Virtual Learning Environment (VLE). This enables the analysis of student behaviour, represented by their actions. The dataset contains the information about 22 courses, 32,593 students, their assessment results, and logs of their interactions with the VLE represented by daily summaries of student clicks (10,655,280 entries). The dataset is freely available at https://analyse.kmi.open.ac.uk/open_dataset under a CC-BY 4.0 license.

## Background & Summary

With the rapid advancement of information technologies, the higher education sector experienced a massive increase in the amount of student data collected. In addition, Virtual Learning Environments emerged and moved courses to the Internet. This transfer was further supported by the boom of Massive Open Online Courses (MOOCs). In the past decade over 200 scientific studies investigated the impact of student data analysis^1^. This shows the importance of open datasets, which provide a standardised way to present and compare results.

To the best of our knowledge, two other open datasets for Learning Analytics exist. The first is the KDD Cup 2010 dataset^2^, which provides data in the form of interaction records between students and a computer-aided-tutoring system. The second dataset is the KDD Cup 2015 dataset^3^ extracted from XuetangX MOOC platform. This dataset contains data about the structure of 40 courses and interactions between students and VLE. It does not include any demographic and historical data from past courses.

In comparison to the above-mentioned datasets, the Open University Learning Analytics dataset (OULAD) contains a subset of the OU student data from 2013 and 2014. It includes both student demographic data and interaction data with the university’s VLE.

To better understand the data the description of the OU and its learning and teaching system follows. The Open University is one of the largest distance learning universities worldwide. At present, around 170,000 students are registered in different programs. Teaching materials and other content are delivered to students via the VLE. Students’ interactions with the educational materials are recorded and stored in university data warehouse.

At the OU, courses are called modules. Modules can be presented multiple times during the year. To distinguish between different presentations of a module, each presentation is named by the year and month it starts. For example, presentations starting in January ends with A, in February with B and so on; so that ‘2013J’ means that the presentation started in October 2013.

The university offers several hundred modules. Each of them can be studied as a stand-alone course or as part of a university programme. No previous qualifications are required.

During the admission process, students are informed about the Data Protection Policy and Policy on Ethical use of Student Data for Learning Analytics. They cover the essential information regarding of use of their personal data. The students are informed that they data are used for academic research purposes and they might be shared with other researchers. In addition, the OU promises not to release data, which might be used to identify individual students, this is not the case of OULAD since this dataset is anonymised and cannot be used to link data with the individual students. At present, the OU does not provide the possibility to opt-out of data usage for Learning Analytics research.

Students in a module-presentation are organised into study groups of approximately 20 people. Each group has an assigned tutor, who guides and supports students throughout the module presentation.

**Figure 1:**
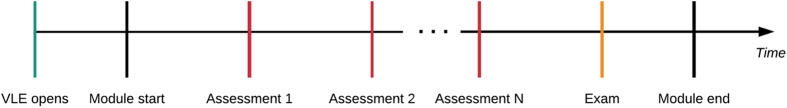
Typical module structure. Module-presentation content is usually available in VLE couple of weeks before official module start. During the presentation the students’ their knowledge is evaluated in series of assessments, which defines the milestones in the module. At the end, there is usually the final exam.

Figure 1 depicts the typical structure of one module-presentation. The typical length of presentations is 9 months. Module resources are available from the VLE system a few weeks before the start of the presentation. Students can sign-up for the module from a few months before the start of the presentation until two weeks after the official module start date. Each module includes several assessments. At the end of the module, there is usually a final exam.

**Figure 2:**
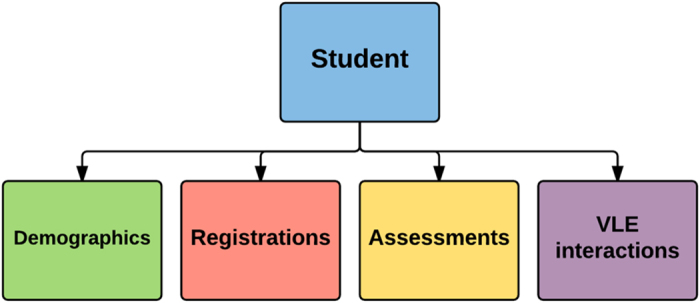
Overall dataset structure. The student is linked with the information about his/her demographics and registrations for the modules. For each student-module-presentation triplet, the dataset contains the results of the students’ assessments and logs of student interactions with VLE.

As stated before the OULAD is a collection of tabular student data from years 2013 and 2014. Each table contains different information, which can be linked to data from other tables using identifier columns. The data contained in the dataset are structured as shown in Fig. 2. The dataset is student oriented, thus the student is the central point. Students’ data includes information about their demographics and registrations for the modules. For each student-module-presentation triplet, the dataset contains the results of the students’ assessments. Student interactions with the VLE are logged as a summary of their daily activities. The dataset contains 22 module-presentations with 32,593 registered students and it is freely available at https://analyse.kmi.open.ac.uk/open_dataset. OULAD has been certified by the Open Data Institute (http://theodi.org/).

The dataset may be used in various scenarios. It enables evaluation of predictive models for predicting student assessment results and final course results and comparison of models with other models develop by other researchers. The VLE data enable to study course structure from the learning perspective and the data itself can be used to evaluate the influence of VLE on the learning outcomes.

## Methods

**Figure 3:**
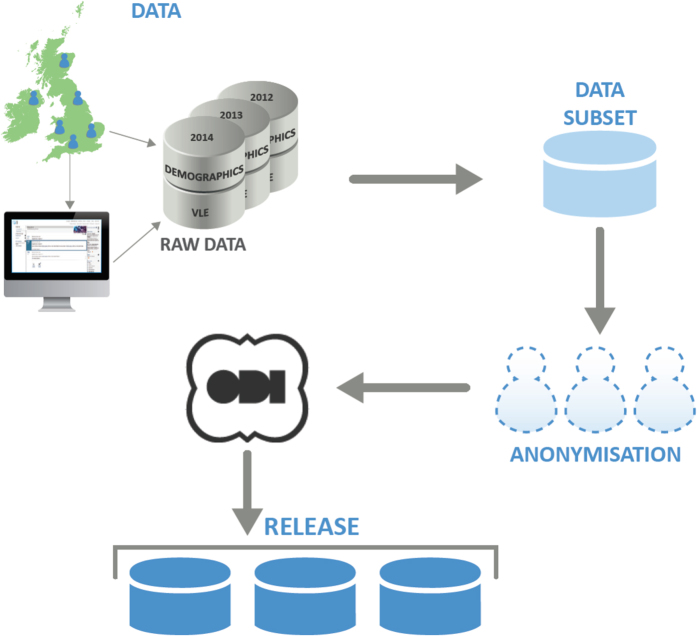
Dataset preparation process. Data were extracted from the OU data warehouse. Then the selected subset has been anonymised, certified by the Open Data Institute and released.

This section describes the stages of preparing the dataset: collection, selection, anonymisation and release. The whole process is depicted in Fig. 3.

### The open university data collection process

At the OU, various information systems for student and module support exist. Due to variability in information collected within each system, the OU implemented a data warehouse, which aggregates information from all available systems. The warehouse is built using SAS technology (https://www.sas.com).

In general, we distinguish three different data types:

- Demographic—represents the basic information about the students including their age, gender, region, previous education, etc.
- Performance—reflects students’ results and achievements during their studies at the OU.
- Learning behaviour—is the log of student activities in the VLE.

### Data selection

The data warehouse contains information about students, namely: their demographics, modules and VLE activities, since 2012. We selected several representative modules taught at the OU during 2013 and 2014. The selection process followed these rules:

- The number of students in the selected module-presentation is larger than 500.
- At least two presentations of the module exist.
- VLE data are available for the module-presentation (since not all the modules are studied via VLE).
- The module has a significant number of failing students.

Out of the all modules that satisfy these criteria we selected 7 modules: 4 Science, Technology, Engineering, and Mathematics (STEM) modules and 3 Social Sciences modules. The total number of students in the selected modules is 38,239.

### Data anonymisation

The dataset anonymisation process was designed according to the ethical and privacy requirements applied at the OU. The whole process of dataset creation and release was supervised by the OU management, and approved by the Vice-Chancellor Executive committee. Anonymisation itself was performed in a series of steps. The first step removed private information about students and modules. This includes the social security number, dates of birth and unique identifiers used at the OU for students. Module names have been replaced by semantic-free symbols and all temporal information has been expressed in relative terms with respect to the presentation start. In addition, all numeric identifiers (i.e., student_id, code_module, etc.) have been reassigned and completely randomised.

Next, we identified quasi-identifying attributes^4^. These are:

- Gender,
- Index of multiple deprivation band (IMD band)^5^,
- Highest education level,
- Age,
- Region, in which student lives, and
- Disability.

These attributes could be used to identify a person using other publicly available sources, thus the necessity to preserve anonymity requires the application of additional anonymisation methods. For that purpose, we used the ARX anonymisation tool^6^, which is widely used in medical domains. Using the information and expert knowledge in the domain we constructed an anonymisation hierarchy for each quasi-identifying attribute, and then applied several anonymisation rules on the dataset using ARX.

The main measure of anonymity we selected was the k-anonymity measure with *k* set to 5. We set the suppression limit to 0.7, this means that the ARX tool prefers to remove ‘outliers’ more often than anonymisation of the quasi-identifier. We also set a maximum number of removed ‘outliers’ to 20 % of all record entries. The last parameter to be set is average re-identification risk criterion, which is set to 0.05. The anonymisation process reduced the number of students to 32,593 and generalised the Age and IMD band attributes.

### Code availability

For the anonymisation we used the ARX anonymisation tool (http://arx.deidentifier.org/) version 3.2.1.

## Data Records

Dataset (Data Citation 1) is available as a set of separate CSV files (comma separated values, each value is within quotation marks and the first line represents column names). Each file contains one ‘database’ table. Tables can be connected using unique identifiers (columns).

**Figure 4:**
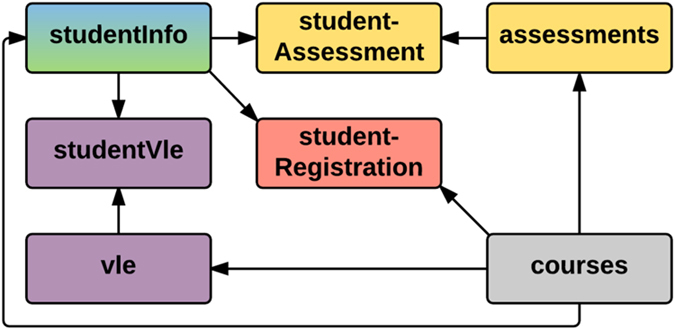
Detailed dataset structure. Table studentInfo is linked to vle courses and assessments table via coresponding "student" table.

Figure 4 shows the detailed structure of the dataset.Table **studentInfo** can be linked to **studentAssessment**, **studentVle** and **studentRegistration** tables using column *id_student*. Table **courses** links to the **assessments**, **studentRegistration**, **vle** and **studentInfo** using identifier columns *code_module* and *code_presentation*. Finally **assessments** table links to **studentAssessment** using *id_assessment* and **vle** to **studentVle** using *id_site*.

The following subsections describe each table in detail. The column identifiers are highlighted using italics.

### Table studentInfo

This table contains student demographic information and also their results in each module they studied. It consists of 32,593 rows with the following columns:

- *code_module*—module identification code on which the student is registered.
- *code_presentation*—presentation identification code during which the student is registered on the module.
- *id_student*—the unique student identification number.
- gender—student’s gender.
- region—the geographic region, where the student lived while taking the module-presentation.
- highest_education—the highest student education level on entry to the module presentation.
- imd_band—the IMD band of the place where the student lived during the module-presentation.
- age_band—a band of student’s age.
- num_of_prev_attempts—the number of how many times the student has attempted this module.
- studied_credits—the total number of credits for the modules the student is currently studying.
- disability—indicates whether the student has declared a disability.
- final_result—student’s final result in the module-presentation.

### Table courses

The table contains the list of all available modules and their presentations. It consists of 22 rows with the following columns:

- *code_module*—code name of the module, which serves as the identifier.
- *code_presentation*—code name of the presentation.
- length—the length of the module-presentation in days from module start date to module end date.

The structure of B and J presentations may differ and therefore it is recommended to analyse the B and J presentations separately. The following table (Table 1) contains information about the study domain, the number of students and the number of presentations of each module included in the dataset.

### Table studentRegistration

Contains information about the time when the student registered for the module presentation. For students who unregistered, the date of un-registration is also recorded. It consists of 32,593 rows with the following columns:

- *code_module*—the module identification code.
- *code_presentation*—the presentation identification code.
- *id_student*—the unique student identification number.
- date_registration—the day of student’s registration for the module presentation.
- date_unregistration—the day of student unregistration from the module presentation. Students, who completed the course have this field empty. Students who unregistered have Withdrawal as the value of the final_result in the studentInfo table.

### Table assessments

This table contains information about assessments in module-presentations. Usually, every presentation has a number of assessments followed by the final exam. The table consists of 206 rows with the following columns:

- *code_module*—module identification code, to which the assessment belongs.
- *code_presentation*—presentation identification code, to which the assessment belongs.
- *id_assessment*—assessment identification number.
- assessment_type—a type of assessment. Three types of assessments exist—Tutor Marked Assessment (TMA), Computer Marked Assessment (CMA) and Final Exam (Exam).
- date—information about the cut-off day of the assessment.
- weight—the weight of the assessment. Typically, Exams are treated separately and have the weight equal to 100%; the sum of all other assessments is also 100%.

If the information about the final exam cut-off day is missing, it takes place during the last week of the module-presentation.

### Table studentAssessment

The table contains the results of students’ assessments. If the student does not submit the assessment, no result is recorded. Results of the final exam are usually missing (since they are scored and used for the final marking immediately at the end of the module). It consists of 173,912 rows with the following columns:

- *id_assessment*—the assessment identification number.
- *id_student*—the unique student identification number.
- date_submitted—the day of assessment submission.
- is_banked—the status flag indicating that the assessment result has been transferred from a previous presentation.
- score—the student’s score in this assessment. The range is from 0 to 100. The score lower than 40 is interpreted as Fail. The marks are in the range from 0 to 100.

### Table studentVle

The studentVle table contains information about student’s interactions with the VLE. It consists of 10,655,280 rows with the following columns:

- *code_module*—the module identification code.
- *code_presentation*—the presentation identification code.
- *id_student*—the unique student identification number.
- *id_site*—the VLE material identification number.
- date—the day of student’s interaction with the material.
- sum_click—the number of times the student interacted with the material.

### Table vle

The vle table contains information about the materials available in the VLE. Typically these are HTML pages, pdf files, etc. It consists of 6,364 rows with the following columns:

- *id_site*—the identification number of the material.
- *code_module*—the identification code for the module.
- *code_presentation*—the identification code of the presentation.
- activity_type—the role associated with the module material.
- week_from—the week from which the material is planned to be used.
- week_to—the week until which the material is planned to be used.

## Technical Validation

OULAD contains data from years 2013 and 2014. We intended to evaluate that the data in the dataset still reflects the current student population. For that purpose, we compared the OULAD data with the corresponding data from 2015. We selected one module, namely CCC, and compared it with the data from 2015. For the comparison, we employed Chi-squared test of goodwill^7^ or Wilcoxon rank sum test^8^ (depends on type on attribute) and applied the selected test on attributes from the studentInfo table. The null hypothesis of the test is whether the distributions of 2013/4 and 2015 data are the same against the alternative hypothesis that distributions are different. To support the results of statistical testing we also produced histograms for visual comparison.

Summary results are depicted in Table 2. We can observe that *P*-value ranges from 0.15 to 0.93 concluding that there is no statistically significant difference between distributions of OULAD and testing data. This leads to the conclusion that dataset is still actual and reflect the current student population well.

**Figure 5:**
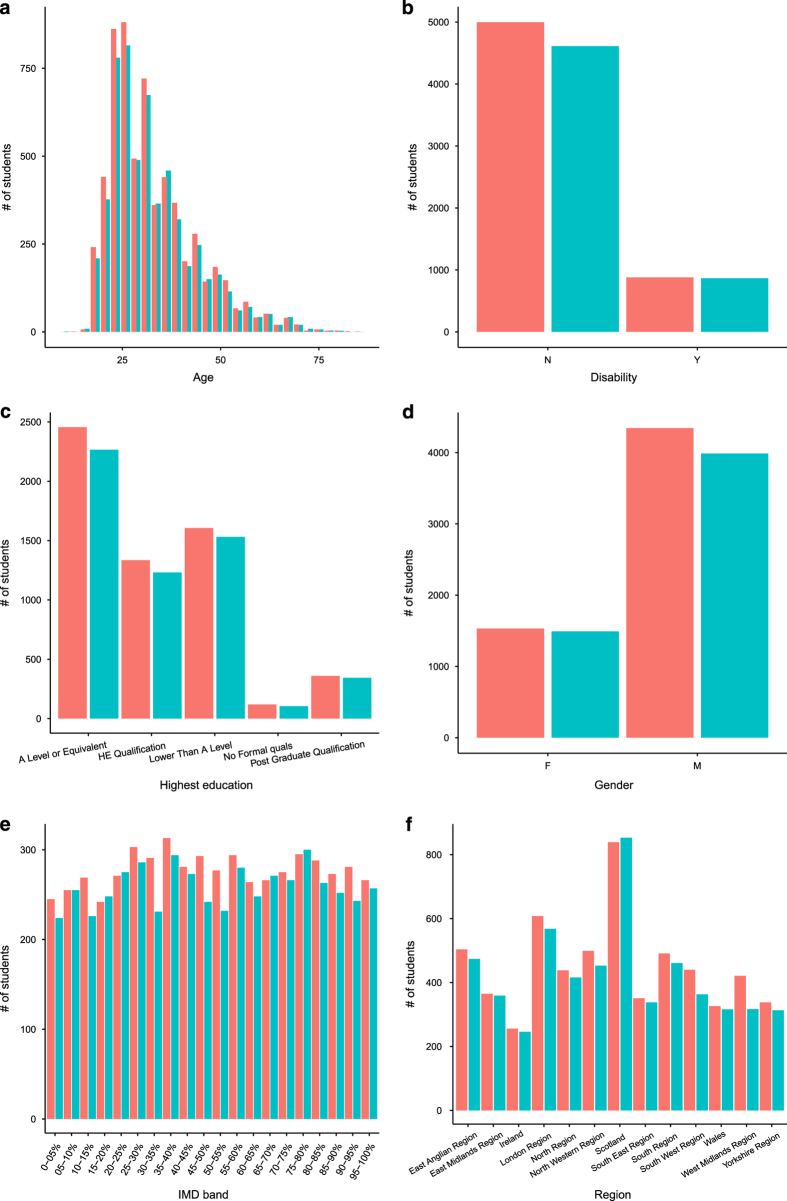
Comparison of attributes of OULAD (red) and testing data (blue) from year 2015. We can observe slight differences between OULAD CCC module and corresponding data from the year 2015. Most of them are caused by a different number of students registered for module each year. The largest difference can be observed in IMD, which is expected since IMD is correlated with the postcode of student home, which changes rapidly.

Figure 5 presents the comparison of attributes of module CCC from the OULAD dataset and the data from the year 2015. We can observe slight differences in data. Most of them are caused by a different number of students studying each year. What is clear from the picture is that the distributions of parameters are similar. The largest difference (in the histogram) can be observed in IMD. This variable is correlated with the postcode of student home, thus it changes rapidly. Nevertheless, the statistics show, that there is no significant difference between OULAD and the testing data, leading to conclusion that the dataset is still up-to-date regarding the student population.

## Usage Notes

We recommend the user to visit the section Examples on OULAD webpage, which contains an example of the dataset usage. The OULAD dataset is also available from the UCI machine learning repository(http://archive.ics.uci.edu/ml/) as a CSV file and from GitHub(http://github.com/) as a R package, which can be installed directly into R using the command: devtools::install_github(‘jakubkuzilek/oulad’).

## Additional Information

**How to cite this article:** Kuzilek, J. *et al.* Open University Learning Analytics dataset. *Sci. Data* 4:170171 doi: 10.1038/sdata.2017.171 (2017).

**Publisher’s note:** Springer Nature remains neutral with regard to jurisdictional claims in published maps and institutional affiliations.

## Supplementary Material



## Figures and Tables

**Table 1:** Module summary and domain information.

**Module**	**Domain**	**Presentations**	**Students**
AAA	Social Sciences	2	748
BBB	Social Sciences	4	7,909
CCC	STEM	2	4,434
DDD	STEM	4	6,272
EEE	STEM	3	2,934
FFF	STEM	4	7,762
GGG	Social Sciences	3	2,534

**Table 2:** Evaluation of similarity between OULAD and 2015 data for CCC module.

**Attribute**	**Test**	**Degrees of freedom**	**Test value**	***P*****-value**
Age	Wilcox	—	17,606,000	0.3558
Disability	*χ*^2^	1	1.0739	0.3001
Education	*χ*^2^	4	0.89201	0.9257
Gender	*χ*^2^	1	2.0537	0.1518
IMD	*χ*^2^	19	15.912	0.6631
Region	*χ*^2^	12	16.325	0.1768
